# Placebos as a Source of Agency: Evidence and Implications

**DOI:** 10.3389/fpsyt.2019.00721

**Published:** 2019-10-25

**Authors:** Phoebe Friesen

**Affiliations:** Biomedical Ethics Unit, Social Studies of Medicine, McGill University, Canada

**Keywords:** placebo effect, deception, agency, expectancy, conditioning, open-label treatments, psychosomatic conditions

## Abstract

Bioethical discussions surrounding the use of placebos in clinical practice have long revolved around the moral permissibility of deceiving a patient if it is likely to benefit them. While these discussions have been insightful and productive, they reinforce the notion that placebo effects can only be induced through deception. This paper challenges this notion, looking beyond the paradigmatic clinical encounter involving deceptive placebos and towards many other routes that bring about placebo effects. After briefly describing the bioethical terrain surrounding the deceptive use of placebos in clinical practice, section 1 offers an examination of the various mechanisms known to contribute to placebo effects: classical conditioning, expectations, affective pathways, open-label placebo treatments, and additional factors that do not fall easily into a single category. The following section explores how each of these routes can be harnessed to bring about clinical benefits without the use of deception. This provides grounding for reconceiving of the placebo effect as a clinical tool that is not always in conflict with patient autonomy and can even be seen as a source of agency. In the final section, implications of the shift away from seeing placebos as necessarily deceptive are discussed. These include the necessity of looking beyond the clinical encounter and mainstream medicine as the primary sites of placebo responses, how important acknowledging the limits of placebo effects will be when we do so, as well as the difficulties of disentangling agency, responsibility, and blame within medicine.

“The placebo, as traditionally used, could be called the lie that heals. But a satisfactory understanding of the nature of the placebo effect shows that the healing comes not from the lie itself, but rather from the relationship between healer and patient, and the latter’s own capacity for self-healing *via* symbolic and psychological approaches as well as *via* biological intervention” (1)

## Introduction: “The Lie That Heals”

Discussions of the placebo effect in clinical practice have long contended with themes of deception, paternalism, and violations of autonomy. In 1907, Richard Cabot (2) argued that “every placebo is a lie, and in the long run the lie is found out”. Arnold et al. (3) described the state of play more recently: “Conscious, deliberate, or incidental/unwitting utilization of the placebo effect is characterized as deceptive, unethical, unscientific, and unprofessional.” Similarly, Kolber (4) reports on how placebo treatments are referred to by some as medicine’s “dirty little secrets.” In line with these associations, most bioethical discussions of the placebo effect revolve around the moral permissibility of using deception within the clinical encounter if it is likely to benefit the patient.^1^ A great deal has been written on this topic, examining the conflict that arises between two central values within medicine, autonomy and beneficence, and weighing the harms and benefits that fall out of prioritizing one over the other (5–9).

Many have defended deception within the clinical encounter. Kihlbom (10) and Shaw (11) have argued that a limited version of consent, which can maximize the benefits of placebos, is sufficient while Barnhill (12) has defended a view in which informed consent and deceptive placebo use need not be seen as incompatible [drawing on (13)]. Miller and colleagues, and later, Alfano (14), have argued that deception is permissible as long as patients consent to it first (sometimes called “authorized deception”) (15,), while Kolber (4) has defended deception on the basis of evidence that patients would prefer to benefit than to be told the truth. On the other side of the debate, many have focused on the harms that might result from the deceptive use of placebos within clinical practice. Blease (17) has suggested that asking patients to authorize deceptive placebo treatments might, paradoxically, lead to worse outcomes by way of nocebo effects^2^, while Asai and Kadooka (18) argue that “the clinical use of placebo and its acceptance would encourage undesirable labeling and contempt for the patient.” Others point out that deceptive placebo use threatens trust and therefore care (19, 20). As Golomb (21) has noted, “The willful breach of trust by doctors to patients on a policy basis may corrode not just *that* physician’s relationship with *that* patient, but may tarnish the reputation of all physicians as trustworthy purveyors of medical advice—abrogating all physicians’ effectiveness, always.”

More recently, bioethical discussions of placebos and deception have also focused on the nocebo effect, asking whether information regarding potential negative side effects of a treatment should be withheld from a patient during informed consent if providing that information makes it more likely that the patient will experience negative side effects^3^ (24–27). While closely related to the conflict that arises between beneficence and autonomy when deceptive placebos are prescribed, this discussion changes tack ever so slightly, examining the tension between nonmaleficence (the avoidance of harm) and patient autonomy.^4^ Proposed solutions include authorized concealment (8, 15), tailoring the informed consent process to the individual (26), and taking into account the specificity and likelihood of each patient’s potential nocebogenic symptoms (25, 27).

Bennet Foddy offers a defense of deceptive placebo use which relies on the description of several cases in which deceptive placebo use is portrayed as the least bad option. These cases include an individual who experiences an improvement in depressive symptoms even though they have been prescribed an ineffective dose of an antidepressant, a patient who has irritable bowel syndrome (IBS) which lacks effective treatments and is responsive to placebo treatments, and a clinician working in a warzone where there are no available treatments. In these cases, Foddy argues, deceptive placebo use is recommended. Since it is the best treatment available, he suggests, it involves “a type of deception that patients ought to be thankful for, just as we are thankful when we receive a mendacious compliment from a friend” (6).

While Foddy may be right that the least bad option is often the best one, it is not clear that any of the cases he presents require deception in order to produce placebo responses. As a result, the least bad option might not be deceptive placebo use, but honest and open placebo use. As I hope to demonstrate below, Foddy and many others who have engaged in bioethical discussions surrounding the deceptive use of placebos have limited themselves to a narrow subset of cases involving the placebo effect. These cases all take place within the clinical encounter and involve a doctor lying to her patient in order to bring about positive expectations surrounding treatment outcomes. If we follow the evidence, however, and examine the myriad ways in which placebo responses are produced, it is no longer obvious that deceptive placebo use ought to take center stage. Rather, placebos emerge as a promising tool for promoting patient autonomy, not merely violating it. In line with this, I make the case below that we should reconsider the age-old association between placebos and deception and examine instead the many ways in which placebos can enhance agency. Agency, in this case, can be thought of as the capacity to act which, in cases of non-deceptive placebo use, results from an increase in available routes by which suffering can be relieved. This capacity can be contrasted with the loss of agency that accompanies dishonest placebo prescriptions, in which patients are unaware of their choices regarding their medical care.

In the next section, I will briefly describe what we know about the mechanisms underlying placebo responses. Building on this evidence base, in the following section, I will argue that there are many ways in which placebo responses can be produced without the use of deception and that non-deceptive routes of placebo intervention ought to be seen as tools that can support the agency of patients. Finally, I will discuss several implications, and the ethical questions surrounding them, that fall out of conceiving of placebo effects as a source of agency.

## What We Know: The Production of Placebo Responses

While defining the placebo effect is inevitably a contentious task, there is some agreement within the field of placebo studies about how placebo responses are produced.^5^ Different theorists tend to place different boundaries around what counts as a placebo effect and divide what falls within those boundaries into different categories. These boundaries and categories are shaped both by empirical evidence and decisions made by theorists. These decisions, in different cases, are informed by ordinary language use, pragmatic arguments, aesthetic appeal, or desires to conserve or break with the past. Here, resting on both empirical evidence and pragmatism, I have divided up the evidence related to placebo effects in a way that will help demonstrate the role they might play in enhancing agency. Below, I briefly discuss what we know of placebo responses brought about by 1) classical conditioning, 2) expectations, 3) affective pathways, 4) open-label placebo treatments, and 5) additional factors, before going on to link these categories to agency in the following section.

While this may become clear in the discussion of evidence that follows, it is worth noting at the outset that some symptoms and conditions are much more responsive to placebo treatments than others. These include pain, both acute and chronic, mood and anxiety disorders, psychogenic movement disorders, autoimmune disorders, and functional somatic syndromes, many of which also lack effective treatments (30–37). Viruses and tumors do not appear to be impacted by placebo treatments, although related symptoms such as hot flashes, fatigue, and nausea, often are (38, 39).

### Classical Conditioning

The role of classical conditioning in bringing about placebo effects has long been recognized (40). Classical conditioning involves the repeated pairing of two stimuli until the result ordinarily produced by one begins to be produced by the other [e.g., Pavlov’s famous experiment in which the sound of a bell produces salivation in a dog after being paired with food enough times (41)]. Conditioned placebo responses have been documented within the endocrine and immune systems and do not appear to be impacted by conscious beliefs or expectations (42). For example, the repeated pairing of cyclophosphamide with anise-flavored syrup led to a reduction of white blood cells (the usual result of cyclophosphamide) merely in response to anise-flavored syrup (42). Prior experience also appears to have a significant impact on analgesic (pain reduction) placebo responses, which can last several days, although, at least in acute cases of pain, these conditioned responses appear to be canceled out by negative expectations of an increase in pain (43, 44).

### Expectations

Expectations, which can be shaped by verbal manipulations, patient beliefs, or contextual factors, appear to impact placebo responses across a variety of symptoms and experiences, including, but not limited to, acute and chronic pain, nausea, inflammation, asthmatic reactions, and motor control (43, 45–47). The role of expectations is evidenced by research that demonstrates that analgesic treatments are significantly more effective when patients are told they are receiving them (as opposed to being administered intravenously and activated from another room) (43, 48). Relatedly, in clinical trials involving treatments for major depressive disorder, the higher the chances of participants receiving the active intervention (trials with more active arms), the more placebo responses occur (49). Similarly, when patients believe they are likely to benefit from a treatment, they are more likely to. In a trial in which participants with low back pain received either massage or acupuncture, their expectations related to treatment had more predictive value related to their outcomes than the treatment they received; those with high expectations benefitted much more (50).

### Relational Components

A significant body of research has also documented the importance of the therapeutic alliance in bringing about placebo responses. In one experiment, patients with a common cold who rated their practitioner as high in empathy were found to have colds that were shorter in duration and less severe than those who perceived less empathy; these patients were also found to have increased immune responses (51). Perceptions of warmth and competence in a practitioner have also been found to progress healing, as evidenced by reduced allergic responses in patients who rated their practitioners as having these qualities (52). Two experiments, one involving patients with IBS and another involving patients with chronic low back pain, both found that additional time and support within the clinical encounter led to significant positive changes in patient outcomes (53, 54). There is also evidence for a correlation between high patient ratings of trust in their practitioner and improved clinical outcomes (55). A growing body of evidence is beginning to unpack why and how we have evolved to be so responsive to empathy, compassion, and those designated healers in our communities, as well as the neural and physiological mechanisms underlying these responses (56).

### Open-Label Placebo Treatments

Growing research on open-label placebo treatments suggests that even when patients are told that they are taking placebo pills which contain no active ingredients, such treatment can lead to significant improvements. This has been demonstrated in patients with IBS, migraines, allergic rhinitis, chronic low back pain, and children with attention-deficit/hyperactivity disorder^6^ (57–61). In one of these trials run by Kaptchuk et al., participants with IBS were recruited and randomized to receive either no treatment or an open-label placebo. Those in the open-label condition took two placebo pills each day and were instructed to think about the potential power of placebo effects. At the end of 3 weeks, these patients scored significantly higher than the no treatment control group on measures of both quality of life and symptom reduction (58). It is not clear what the mechanisms behind open-label placebo responses are. While recent reviews of the phenomenon have suggested that classical conditioning, expectations, and social support may all contribute (62, 63), others have suggested that these explanations are insufficient and that theories of embodied cognition and Bayesian predictive processing might better account for the success of open-label treatments (64).

### Additional Factors

There are also several sources of placebo responses where the mechanisms at work are still unclear. Possibly linked to expectation-based placebo responses is evidence that suggests that placebo effects increase when one is given a choice of what analgesic to take (65), when a treatment is thought to be expensive (66), and when a treatment is invasive (67–69). Conditioning might explain greater placebo responses being derived from more frequent interventions (70, 71) or greater adherence to a treatment (72), while relational components may contribute to better outcomes in patients with nonspecific chest pain who received more diagnostic tests, despite these tests having no impact on treatment (73). Social learning (e.g., watching another person experience pain relief from a particular treatment) also contributes to analgesic placebo responses, which could be a result of either expectations or conditioning (74).

## An Alternative View: Placebos as a Source of Agency

In a discussion of the role of the placebo effect in clinical practice, Alfano (14) acknowledges that “deception is not required to alter a patient’s expectations, to classically condition them, or to modulate their somatic attention,” and yet, he recommends the use of authorized deception and concealment within the clinical encounter. He argues that obtaining consent to deceive patients will contribute to increases in placebo responses through expectations and encourage greater adherence, promoting conditioned placebo responses. Such recommendations emphasize the importance of deception in bringing about placebo effects, promoting a picture that fails to recognize how placebo effects can be brought about without dishonesty. What if, rather than focusing on how deception can bring about placebo responses, we looked to the ways in which placebo effects can be used in conjunction with patient autonomy? In this section, following from the placebo pathways presented in the previous section, I will demonstrate how each of these can be manipulated in order to enhance agency rather than deny it.

### Classical Conditioning

Placebo responses brought about by way of classical conditioning have little need for deception, as a result of their tendency to remain disconnected from cognitive processes. In particular, conditioning can be used to enhance an existing therapeutic response and, in some cases, to reduce one’s medication dosage in order to avoid side effects while maintaining the same level of efficacy (34, 39, 75). Dose reduction *via* placebo conditioning has been demonstrated to be effective with antihistamines for allergic reactions, methadone for those with opioid use disorder, melatonin for children with difficulties sleeping, antipsychotics for individuals diagnosed with schizophrenia, and corticosteroids for the treatment of psoriasis (76–80, 81).^7^ This suggests that classically conditioned placebo responses can be used to support tapering or weaning off a medication entirely, opening up new avenues for patients for which treatments are effective but cannot be sustained. Some groups that might benefit from such conditioned placebo responses include those who are unable to afford a medication, those who wish to taper their dose of a treatment because of negative side effects, or individuals with complex pharmaceutical regimens who hope to avoid adverse interactions between drugs (83). As mentioned above, awareness of the conditioning process does not appear to impact conditioned immune and endocrine responses, so there is no need for deception. This is slightly more complicated in the case of pain, where negative expectations appear to overrule positive classical conditioning that has come before. This suggests that, at least with regards to acute pain, conditioned placebo responses may need to be generated along with expectation-based placebo responses.

### Expectations

Expectation-based placebo responses are a more complicated case, in that deceptive placebo use is primarily based on intentions to manipulate patient expectations. However, such an approach to placebo use assumes that one’s expectations related to one’s clinical outcomes are entirely created within the clinical context. While the doctor’s words may have a significant impact on what one anticipates, many other sources outside of the doctor’s office contribute to shaping patient expectations as well. These sources include, but are not limited to, past experiences, information that one has read online, stories one has been exposed to about similar cases, related narratives in the media and popular culture, and what friends and family members have led one to expect. For example, joining a support group of individuals who have learned to live well despite the presence of chronic pain may alter one’s expectations of one's own pain, leading to a reduction in suffering. As a result, individuals who are struggling with the kinds of symptoms and conditions that tend to be placebo responsive can actively shift their own expectations through exposing themselves to particular information and narratives, which are more likely to produce placebo, rather than nocebo, responses in themselves. Similarly, they can choose treatments that they believe are likely to work and that align with their values, thereby increasing the chances that they will (84).

### Relational Components

Deception and violations of autonomy are certainly not required to produce placebo responses in patients by way of relational components like warmth, empathy, and trust. These fall naturally out of positive clinical encounters. Efforts can be made to spend more time with patients and listen to them more carefully, as these are likely to increase placebo responses, particularly in those conditions that tend towards robust placebo effects. Furthermore, while there is a great deal of placebo literature focusing on the impact of aspects of the clinical encounter, it may be that these benefits can be gained through other social encounters as well. There is ample evidence that social support makes a difference to many clinical outcomes, particularly those related to mental and cardiovascular health (85–87). It is unclear whether warmth, trust, and empathy, in the clinical encounter, lead to improvements in wellbeing through the same mechanisms that warmth, trust, and empathy, outside of the clinical encounter do, but it is worth cashing in on both avenues. If research suggests that both a positive clinical encounter and a few hugs a day (see 88) are likely to be protective against illness, this suggests that there are multiple routes by which individuals can seek to boost their own placebo responses through supportive relationships.

### Open-Label Placebo Treatments

Open-label placebo treatments are probably the most obvious way in which placebo responses can be harnessed without the use of deception because they involve a complete disclosure that the treatment is a placebo. While the evidence base is still quite limited, the research that does exist suggests that these treatments hold promise. The diversity of conditions that have been found to improve through open-label placebo treatments indicates that there may be many more worth exploring; as mentioned above, these include IBS, migraines, allergic rhinitis, and chronic low back pain. As with conditioned placebo responses, those who may be most likely to benefit may be individuals who cannot afford ordinary treatments, those who require polypharmaceutical regimens, or those who experience significant side effects from a particular treatment.

### Additional Factors

Finally, the grab bag of routes that appear to lead to placebo effects, but that we do not currently understand well, is likely to offer additional tools by which individuals can benefit from placebo responses without the use of deception. If more invasive treatments appear to lead to better outcomes than noninvasive ones, then perhaps pairing a particularly pungent drink with one’s medication or treatment can be of value. If frequency of treatment and adherence to a treatment also impact clinical outcomes, patients can divide pills into smaller doses to increase frequency and use reminders to increase their adherence in order to tap into these potential increases in efficacy. Similarly, if social learning contributes to placebo responses, exposing oneself to success stories of individuals who have recovered from a similar experience may be worthwhile.

## Implications: Advancing Bioethical Discussions of Placebos

As evidenced above, the link between deception and placebo treatments is not a necessary one. Placebo effects are produced through many avenues which can be harnessed through nondeceptive means. Acknowledging these routes of placebo intervention is likely to advance bioethical discussions of placebo effects beyond questions concerning the moral appropriateness of dishonesty for the sake of clinical benefit. While considering the conflict that arises between beneficence and autonomy during deceptive placebo use is an important ethical issue, it is not the only issue pertinent to discussions of placebo treatments within medical ethics. In this section, I discuss four implications that fall out of shifting away from focusing on placebo treatments as associated with deception and towards seeing placebos as a source of agency. These implications raise new ethical questions that appear on the placebo landscape once we look beyond deceptive use, some of which I flag within the discussion below.

### Looking Outside the Clinical Encounter

The first implication is that recognizing the role of agency in placebo effects takes us beyond the clinical context and requires us to see the potential for promoting placebo effects in several other realms. Rather than thinking only of the question of whether doctors should lie to patients for their medical benefit, examining the mechanisms underlying placebos and how they can promote agency reveals the significant role that placebo effects play in many domains of our lives. Many have pushed towards expanding the boundaries of the sources of placebo effects before. Miller and Kaptchuk (89) have suggested that “instead of focusing exclusively on the therapeutic power of medical technology and thereby ignoring or dismissing context, we should see the context of the clinical encounter as a potential enhancer, and in some cases the primary vehicle, of therapeutic benefit.” Even beyond the context of the clinical encounter, however, there are routes by which expectations are shaped, associations are created, and relationships may contribute to placebo responses. Narrowing in on the mechanisms by which placebo responses are created leads one to recognize the significant roles that nonmedical contexts (e.g., online spaces, workplaces, schools) and nonmedical people (e.g., friends, family, characters) are playing in shaping both placebo and nocebo effects. For example, if social support and empathy bring about placebo responses for many conditions, it is crucial that we look to the networks and relationships individuals are embedded in as a source of placebo effects, as well as what happens in the doctor’s office. Of course, such networks and relationships, or a lack thereof, can also be the source of nocebo effects.

Shifting our attention outside of the clinical encounter and towards other spaces in which placebo responses are likely to be generated allows us to see many more settings and influences that are relevant to discussions of the placebo effect. Rather than merely focusing on the doctor’s office, we can begin to examine the role of individual and collective rituals and stories, social settings and communities one partakes in, and the many relationships one is embedded in, in producing placebo responses. Evidence related to “placebo by proxy” supports this extension, demonstrating, perhaps unsurprisingly, that sometimes placebo effects in children may be mediated more by their parents than by their doctor (90). Looking beyond the white walls and white coat leads to difficult ethical questions related to what falls within the bounds of medicine and what the responsibilities of health-care professionals might be in relation to placebo responses that take place outside of their territory. If it is the case that many factors that may influence placebo responses are outside of the health-care system, is there a responsibility to communicate with patients about these influences within the process of informed consent? If so, what should they be told? Should the sources of placebo effects merely be prescribed, and a warning given, or should recommendations regarding how to enhance placebo effects and avoid nocebo effects be offered? Furthermore, does recognizing the wider scope of placebo influences have implications for how patient support networks should be run or for potential additional variables that ought to be controlled for within clinical trials?

### Looking Outside Mainstream Medicine

Broadening our examination of the territory of placebo phenomena also allows us to look beyond mainstream or Western medical contexts, which provide the setting for a great deal of placebo research. Given the mechanisms underlying placebo responses, it seems likely that practitioners of complementary, alternative, and traditional medicines are likely to be contributing to placebo effects regularly (91, 92). This is because many of the features that tend to enhance placebo responses, particularly in relation to expectations and relational components, tend to show up in these forms of medicine, and because the conditions that people most frequently seek these treatments for are ones that tend to be highly responsive to placebo treatments (93). If evidence suggests that choosing a treatment that aligns with one’s values can enhance placebo responses, what does this mean for treatments that do not fall within the evidence base but that many people would like to receive? How can this be taken into account within systems of evaluating the efficacy of treatments?

Challenging evidence related to these questions comes from a recent examination of the impact of different components of homeopathy on clinical outcomes in patients with rheumatoid arthritis. The findings suggest that whether one takes part in the homeopathic consultation, which is often quite extensive, involves particular attention to the therapeutic alliance, and is likely to generate hope and positive expectations, is more predictive of positive clinical outcomes than whether one receives a homeopathic treatment (94). This raises interesting ethical questions regarding the role that complementary, alternative, and traditional medicines ought to play or not play within health care. One might argue, based on this research, that homeopathy is merely a form of deceptive placebo use, and yet, it is possible that an open-label placebo treatment involving homeopathy would be effective for some people and some conditions. Does this suggest that we should make such treatments more widely available, given the difficulty of finding such elaborate care in mainstream medicine? Or does this mean that practitioners should be required to fully disclose which components of the treatment are likely to be contributing to positive outcomes and which are not? How should medical practices that primarily offer therapeutic effects *via* placebo responses be regulated?

### Acknowledging the Limits of Placebo Treatments

Related to this is the importance of being clear about in which cases there might be room for improvement through the manipulation of placebo response and in which cases there is not. As mentioned above, there are some types of symptoms and conditions that tend to be highly responsive to placebo treatments (e.g., pain, mood, anxiety, psychosomatic symptoms or conditions) while others do not appear to be impacted at all (e.g., viruses, tumors). Unfortunately, there is a risk that acknowledging placebo use as a source of agency could lead to creating, or further cementing, inaccurate beliefs about where placebo treatments can be effective. This could occur if excitement generated about having the ability to impact one’s own wellbeing in one domain bleeds into another domain, leading claims about the success of alternative treatments for IBS and the success of alternative treatments for cancer to be seen as equivalent, when based on what we know about the placebo effect, these two claims ought to be treated very differently.

It is well documented that an interest in alternative medicine aligns with a higher likelihood of refusing conventional therapies for cancer, which is linked to higher mortality rates, and with a greater tendency towards vaccine hesitancy (95–97). To acknowledge that some alternative, complementary, and traditional therapies may be quite effective in treating some symptoms and conditions by way of the placebo effect could indirectly encourage beliefs that all medical problems are treated equally well by such therapies. While there is a significant amount of research yet to be done that will better allow us to demarcate the boundaries of placebo potential, it is important to be as honest as possible at this point about what placebo responses can and cannot do for people. The risks and rewards that are likely to accompany experiences of seeking alternative care for chronic pain look very different from the risks and rewards that are likely to accompany experiences of seeking alternative care for lung cancer. Recognizing these limits raises questions related to how placebo research ought to be responsibly reported in scientific publications and the media, how clinicians working in integrative medicine should communicate with patients and the public about the evidence and mechanisms underlying the treatments they offer and about what research priorities in the field of placebo studies ought to be.

### Enhancing Agency Without Enhancing Blame^8^

Finally, given that an emphasis on individual agency within health conditions often brings with it an attentiveness to individual responsibility and blame, we ought to be careful in exploring the links between placebo effects and agency. Particularly when considering the capacity for individuals to produce nocebo effects, which produce negative rather than positive outcomes, it is crucial that we do not burden individuals with the weight of responsibility and blame for their own suffering (99). This is especially relevant with regards to conditions characterized as psychosomatic, many of which tend to show robust responses to placebo treatment. These conditions, however, are already among the most stigmatized within medicine, in large part because there is a tendency to characterize conditions in which psychological and somatic symptoms interact as less real or as being “all in the head” (100–103). As Greco (104) has suggested, what distinguishes biomedical and psychosomatic conceptions of illness is “a shift from aetiological or causal explanations to explanations that might be termed ‘dispositional’.” This shift leads to an understanding of psychosomatic conditions as associated with an individual’s moral failings, in part because the “perception of a need for medical care is not corroborated by a medical diagnosis based on physio-chemical evidence” (104).^9^

This suggests that we ought to be very careful in embracing the potential of conditioned, expectation-based, open-label, and relational placebo effects in these conditions, in that we do not want to create more stigma, and more harm, by reinforcing notions of blame and moral failing in these patients. Recognizing this tension raises questions about how to best to utilize these tools without directing attention to blame and responsibility. Is it likely that thinking of these routes of intervention as placebo effects will reinforce stigma within these patient populations? How might we better characterize placebo phenomenon so that they can be harnessed while causing the least harm possible? Would we be better off focusing on the individual routes by which outcomes are improved (e.g., conditioning, expectations) rather than thinking of placebo effects as a whole, as suggested by Alfano (14), or throwing out the term entirely, as suggested by Nunn (106)?

A broader version of this concern relates to how noting links between agency and wellbeing can promote healthism, which views health as a private resource that individuals are responsible for securing for themselves (107, 108). If we place the responsibility on individuals to ensure that they harness these agential placebo effects, we may end up alienating them rather than motivating them. Furthermore, not everyone has equal access to the resources that might allow them to benefit from these nondeceptive placebos, including a warm and empathetic clinician, the time and money for reiki, or unlimited hugs.^10^

## Conclusion

The placebo effect has long been associated with deception, lies, and clinical paternalism. While these associations are grounded in common ways in which the phenomenon has been, and continues to be, manipulated in clinical practice, these associations are not inherently linked to the phenomenon. As we learn more about the routes by which placebo effects can be generated, it is becoming clear that deception is not a necessary component of placebo prescription, but an accidental one. Placebo responses can operate by way of conditioning, expectations, relational factors, open-label placebo treatments, and other routes, which do not require a patient to be deceived. Recognizing the diverse ways in which patients can benefit from placebo effects without deception not only allows us to see a much greater potential in the phenomenon but also significantly widens the scope of ethical issues that we must contend with. In this manuscript, I hope to have gestured towards some of the bioethical issues that are likely to arise as the placebo effect continues to shed its “legacy of trickery” and becomes recognized as a powerful phenomenon that does not always need to lie to get its way (110).

## Author Contributions

This manuscript was conceptualized, researched, and written by PF.

## Funding

This research was supported by the National Institute for Health Research (NIHR) Oxford Biomedical Research Centre, grant BRC-1215-20008 to the Oxford University Hospitals NHS Foundation Trust and the University of Oxford. The views expressed are those of the author and not necessarily those of the NHS, the NIHR or the Department of Health.

## Conflict of Interest

The author declares that the research was conducted in the absence of any commercial or financial relationships that could be construed as a potential conflict of interest.

## Footnotes

1 There is also a significant body of bioethical literature concerned with the use of placebo controls in research, but this literature revolves around the placebo as a control rather than as a phenomenon to be harnessed.

2 Nocebo effects are akin to placebo effects but involve negative clinical outcomes rather than positive ones.

3 For example, when men being prescribed finasteride for benign prostatic hyperplasia (prostate gland enlargement) were split into two groups, one of which was warned of potential sexual side effects and one of which was not, 44% of those who were warned reported experiencing sexual dysfunction, compared to only 15% of those who were not warned (22). Such side effects have also been reported as a result of finasteride outside of the research setting, but it is unclear whether they were induced through nocebo mechanisms or not (23).

4 The relationship between beneficence and nonmaleficence is often an ambiguous one. As Veatch has pointed out in relation to the Belmont Report, it is unclear whether &amp;ldquo;beneficence&amp;rdquo; is meant to capture both beneficence and nonmaleficence as two sides of the same coin, or whether they should be seen as distinct values (28).

5 Although for an interesting argument that there is more consensus within the field than is often acknowledged, see (29).

6 Note that conclusions drawn from the trial by Sandler and Bodfish with children with a diagnosis of attention-deficit/hyperactivity disorder should be limited. Teachers, who were blinded, did not find that open label placebo plus a 50% dose of medication was as effective as a 100% dose, while parents and clinicians, who were not blinded, did.

7 There is even evidence in rats that placebo conditioning of heart allografts can prolong transplant survival (82).

8 A nod to Hannah Pickard&amp;rsquo;s useful notion of responsibility without blame (98).

9 This relates to the significant disagreement between patient organizations and medical authorities over the status of myalgic encephalopathy (also known as chronic fatigue syndrome) as a psychological (favored by clinicians) or physical (favored by patients) condition (105) [although see (103) for criticisms of the methodology used by Hossenbaccus and White].

10 This also suggests that some will be least well off when it comes to benefitting from placebo effects, both those arising within the clinical encounter and arising outside of it [see also (109) on this topic].

## References

[1] Brody H The lie that heals: the ethics of giving placebos. Ann Intern Med (1982) 97(1):112–8. 10.7326/0003-4819-97-1-112 7046551

[2] Cabot RC The physician’s responsibility for the nostrum evil. Cal State J Med (1907) 5(1):12–3. 10.1001/jama.1906.25210130006001b PMC1651813 18733994

[3] Arnold MH Finniss DG Kerridge I Destigmatising the placebo effect. Am J Bioethics (2015) 15(10):21–3. 10.1080/15265161.2015.1074312 26479096

[4] Kolber AJ A limited defense of clinical placebo deception. Yale Law Policy Rev (2007) 26(1):75–134.

[5] Bok S The ethics of giving placebos. Sci Am (1974) 231(5):17–23. 10.1038/scientificamerican1174-17 4432060

[6] Foddy B A duty to deceive: placebos in clinical practice. Am J Bioeth (2009) 9(12):4–12. 10.1080/15265160903318350 20013484

[7] Kolber A How placebo deception can infringe autonomy. Am J Bioeth (2009) 9(12):25–6. 10.1080/15265160903242725 20013492

[8] Miller FG Colloca L The placebo phenomenon and medical ethics: rethinking the relationship between informed consent and risk–benefit assessment. Theor Med Bioethics (2011) 32(4):229–43. 10.1007/s11017-011-9179-8 21479794

[9] Barnhill A Miller FG The ethics of placebo treatments in clinical practice: a reply to Glackin. J Med Ethics (2015) 41(8):673–6. 10.1136/medethics-2014-102651 26100361

[10] Kihlbom U Autonomy and negatively informed consent. J Med Ethics (2008) 34(3):146–9. 10.1136/jme.2007.020503 18316453

[11] Shaw DM Prescribing placebos ethically: the appeal of negatively informed consent. J Med Ethics (2009) 35(2):97–9. 10.1136/jme.2008.025700 19181881

[12] Barnhill A What it takes to defend deceptive placebo use. Kennedy Inst Ethics J (2011) 21(3):219–50. 10.1353/ken.2011.0015 22073816

[13] O’Neill O Paternalism and partial autonomy. J Med Ethics (1984) 10(4):173–8. 10.1136/jme.10.4.173 PMC1375094 6520849

[14] Alfano M Placebo effects and informed consent. Am J Bioethics (2015) 15(10):3–12. 10.1080/15265161.2015.1074302 26479091

[15] Colloca L Miller FG The nocebo effect and its relevance for clinical practice. Psychosom Med (2011) 73(7):598. 10.1097/PSY.0b013e3182294a50 21862825 PMC3167012

[16] Miller F. G. Wendler D. Swartzman L. C. (2005). Deception in research on the placebo effect. PLoS medicine, 2(9), e262. 10.1371/journal.pmed.0020262 16173830 PMC1198039

[17] Blease C Authorized concealment and authorized deception: well-intended secrets are likely to induce nocebo effects. Am J Bioethics (2015) 15(10):23–5. 10.1080/15265161.2015.1074310 26479097

[18] Asai A Kadooka Y Reexamination of the ethics of placebo use in clinical practice. Bioethics (2013) 27(4):186–93. 10.1111/j.1467-8519.2011.01943.x 22296589

[19] Powell T Bailey J Against placebos. Am J Bioeth (2009) 9(12):23–5. 10.1080/15265160903244234 20013491

[20] Schwab AP When subtle deception turns into an outright lie. Am J Bioeth (2009) 9(12):30–2. 10.1080/15265160903234128 20013495

[21] Golomb BA Doctoring the evidence: the case against lying to patients about placebos. Am J Bioeth (2009) 9(12):34–6. 10.1080/15265160903244242 20013497

[22] Mondaini N Gontero P Giubilei G Lombardi G Cai T Gavazzi A Finasteride 5 mg and sexual side effects: how many of these are related to a nocebo phenomenon? J Sex Med (2007) 4(6):1708–12. 10.1111/j.1743-6109.2007.00563.x 17655657

[23] Justman S The finasteride riddle. J Symptoms Signs (2014) 3(3):154.

[24] Colloca L Finniss D Nocebo effects, patient–clinician communication, and therapeutic outcomes. JAMA (2012) 307(6):567–8. 10.1001/jama.2012.115 PMC6909539 22318275

[25] Wells RE Kaptchuk TJ To tell the truth, the whole truth, may do patients harm: the problem of the nocebo effect for informed consent. Am J Bioethics (2012) 12(3):22–9. 10.1080/15265161.2011.652798 PMC3352765 22416745

[26] Cohen S The nocebo effect of informed consent. Bioethics (2014) 28(3):147–54. 10.1111/j.1467-8519.2012.01983.x 22762392

[27] Fortunato JT Wasserman JA Menkes DL When respecting autonomy is harmful: a clinically useful approach to the nocebo effect. Am J Bioethics (2017) 17(6):36–42. 10.1080/15265161.2017.1314042 28537834

[28] Veatch RM Ranking, balancing, or simultaneity: resolving conflicts among the belmont principles. In: Childress JF Meslin EM Shapiro HT, editors. Belmont revisited: ethical principles for research with human subjects. Washington, DC: Georgetown University Press (2005). p. 184–204.

[29] Blease C Consensus in placebo studies: lessons from the philosophy of science. Perspect Biol Med (2018) 61(3):412–29. 10.1353/pbm.2018.0053 30293979

[30] Coryell W Noyes R Placebo response in panic disorder. Am J Psychiatry (1988) 145(9):1138–40. 10.1176/ajp.145.9.1138 3046384

[31] Hayden SP A practical approach to chronic fatigue syndrome. Clev Clin J Med (1991) 58(2):116. 10.3949/ccjm.58.2.116 2025913

[32] Vase L Robinson ME Verne GN Price DD The contributions of suggestion, desire, and expectation to placebo effects in irritable bowel syndrome patients: an empirical investigation. Pain (2003) 105(1–2):17–25. 10.1016/S0304-3959(03)00073-3 14499416

[33] Khan A Kolts RL Rapaport MH Krishnan KR Brodhead AE Browns WA Magnitude of placebo response and drug–placebo differences across psychiatric disorders. Psychol Med (2005) 35(5):743–9. 10.1017/S0033291704003873 15918351

[34] Patel SM Stason WB Legedza A Ock SM Kaptchuk TJ Conboy L The placebo effect in irritable bowel syndrome trials: a meta-analysis1. Neurogastroenterol Motil (2005) 17(3):332–40. 10.1111/j.1365-2982.2005.00650.x 15916620

[35] Pacheco-Lopez G Engler H Niemi MB Schedlowski M Expectations and associations that heal: immunomodulatory placebo effects and its neurobiology. Brain Behav Immun (2006) 20(5):430–46. 10.1016/j.bbi.2006.05.003 16887325

[36] Zhang W Robertson J Jones A Dieppe P Doherty M The placebo effect and its determinants in osteoarthritis: meta-analysis of randomised controlled trials. Ann Rheum Dis (2008) 67(12):1716–23. 10.1136/ard.2008.092015 18541604

[37] Rommelfanger KS Opinion: a role for placebo therapy in psychogenic movement disorders. Nat Rev Neurol (2013) 9(6):351. 10.1038/nrneurol.2013.65 23628738

[38] de la Cruz M Hui D Parsons HA Bruera E Placebo and nocebo effects in randomized double-blind clinical trials of agents for the therapy for fatigue in patients with advanced cancer. Cancer (2010) 116(3):766–74. 10.1002/cncr.24751 PMC2815077 19918921

[39] Kaptchuk TJ Miller FG Placebo effects in medicine. N Engl J Med (2015) 373(1):8–9. 10.1056/NEJMp1504023 26132938

[40] Ader R Conditioned immune responses and pharmacotherapy. Arthritis Care Res (1989) 2(3):S58–64. 10.1002/anr.1790020315 2487705

[41] Pavlov IP Conditional reflexes: an investigation of the physiological activity of the cerebral cortex. Ann Neurosci (2010) 17(3): 136–141. 10.5214/ans.0972-7531.1017309 25205891 PMC4116985

[42] Giang DW Goodman AD Schiffer RB Mattson DH Petrie M Cohen N Conditioning of cyclophosphamide-induced leukopenia in humans. J Neuropsychiatry Clin Neurosci (1996) 8(2):194–201. 10.1176/jnp.8.2.194 9081556

[43] Benedetti F Pollo A Lopiano L Lanotte M Vighetti S Rainero I Conscious expectation and unconscious conditioning in analgesic, motor, and hormonal placebo/nocebo responses. J Neurosci (2003) 23(10):4315–23. 10.1523/JNEUROSCI.23-10-04315.2003 PMC6741114 12764120

[44] Colloca L Benedetti F How prior experience shapes placebo analgesia. Pain (2006) 124(1-2):126–33. 10.1016/j.pain.2006.04.005 16701952

[45] Luparello T Lyons HA Bleecker ER McFadden ER Jr. Influences of suggestion on airway reactivity in asthmatic subjects. Psychosom Med (1968) 30(6):819–25. 10.1097/00006842-196811000-00002 5726052

[46] Hashish BI Harvey W Harris M Anti-inflammatory effects of ultrasound therapy: evidence for a major placebo effect. Br J Rheumatol (1986) 25: 77–81. 10.1093/rheumatology/25.1.77 2417648

[47] Benedetti F Lanotte M Lopiano L Colloca L When words are painful: unraveling the mechanisms of the nocebo effect. Neuroscience (2007) 147(2):260–71. 10.1016/j.neuroscience.2007.02.020 17379417

[48] Levine JD Gordon NC Influence of the method of drug administration on analgesic response. Nature (1984) 312(5996):755–6. 10.1038/312755a0 6514008

[49] Papakostas GI Fava M Does the probability of receiving placebo influence clinical trial outcome? A meta-regression of double-blind, randomized clinical trials in MDD. Eur Neuropsychopharmacol (2009) 19(1):34–40. 10.1016/j.euroneuro.2008.08.009 18823760

[50] Kalauokalani D Cherkin DC Sherman KJ Koepsell TD Deyo RA Lessons from a trial of acupuncture and massage for low back pain: patient expectations and treatment effects. Spine (Phila Pa 1976) (2001) 26(13):1418–24. 10.1097/00007632-200107010-00005 11458142

[51] Rakel DP Hoeft TJ Barrett BP Chewning BA Craig BM Niu M Practitioner empathy and the duration of the common cold. Fam Med (2009) 41(7):494–501. 19582635 PMC2720820

[52] Howe LC Goyer JP Crum AJ Harnessing the placebo effect: exploring the influence of physician characteristics on placebo response. Health Psychol (2017) 36(11):1074–82. 10.1037/hea0000499 PMC7608626 28277699

[53] Kaptchuk TJ Kelley JM Conboy LA Davis RB Kerr CE Jacobson EE Components of placebo effect: randomised controlled trial in patients with irritable bowel syndrome. Bmj (2008) 336(7651):999–1003. 10.1136/bmj.39524.439618.25 18390493 PMC2364862

[54] Fuentes J Armijo-Olivo S Funabashi M Miciak M Dick B Warren S Enhanced therapeutic alliance modulates pain intensity and muscle pain sensitivity in patients with chronic low back pain: an experimental controlled study. Phys Ther (2014) 94(4):477–89. 10.2522/ptj.20130118 24309616

[55] Birkhauer J Gaab J Kossowsky J Hasler S Krummenacher P Werner C Trust in the health care professional and health outcome: a meta-analysis. PLoS One (2017) 12(2):e0170988. 10.1371/journal.pone.0170988 PMC5295692 28170443

[56] Benedetti F Placebo and the new physiology of the doctor–patient relationship. Physiol Rev (2013) 93(3):1207–46. 10.1152/physrev.00043.2012 PMC3962549 23899563

[57] Sandler AD Bodfish JW Open-label use of placebos in the treatment of ADHD: a pilot study. Child Care Health Dev (2008) 34(1):104–10. 10.1111/j.1365-2214.2007.00797.x 18171451

[58] Kaptchuk TJ Friedlander E Kelley JM Sanchez MN Kokkotou E Singer JP Placebos without deception: a randomized controlled trial in irritable bowel syndrome. PLoS One (2010) 5(12):e15591. 10.1371/journal.pone.0015591 PMC3008733 21203519

[59] Kam-Hansen S Jakubowski M Kelley JM Kirsch I Hoaglin DC Kaptchuk TJ Altered placebo and drug labeling changes the outcome of episodic migraine attacks. Sci Transl Med (2014) 6(218):218ra215. 10.1126/scitranslmed.3006175 PMC4005597 24401940

[60] Carvalho C Caetano JM Cunha L Rebouta P Kaptchuk TJ Kirsch I Open-label placebo treatment in chronic low back pain: a randomized controlled trial. Pain (2016) 157(12):2766–72. 10.1097/j.pain.0000000000000700 PMC5113234 27755279

[61] Schaefer M Harke R Denke C Open-label placebos improve symptoms in allergic rhinitis: a randomized controlled trial. Psychother Psychosom (2016) 85(6):373–4. 10.1159/000447242 27744433

[62] Petkovic G Charlesworth JE Kelley J Miller F Roberts N Howick J Effects of placebos without deception compared with no treatment: protocol for a systematic review and meta-analysis. BMJ Open (2015) 5(11):e009428. 10.1136/bmjopen-2015-009428 PMC4663432 26610763

[63] Charlesworth JE Petkovic G Kelley JM Hunter M Onakpoya I Roberts N Effects of placebos without deception compared with no treatment: a systematic review and meta-analysis. J Evid Based Med (2017) 10(2):97–107. 10.1111/jebm.12251 28452193

[64] Kaptchuk TJ Open-label placebo: reflections on a research agenda. Perspect Biol Med (2018) 61(3):311–34. 10.1353/pbm.2018.0045 30293971

[65] Rose JP Geers AL Rasinski HM Fowler SL Choice and placebo expectation effects in the context of pain analgesia. J Behav Med (2012) 35(4):462–70. 10.1007/s10865-011-9374-0 21850515

[66] Waber RL Shiv B Carmon Z Ariely D Commercial features of placebo and therapeutic efficacy. JAMA (2008) 299(9):1016–7. 10.1001/jama.299.9.1016 18319411

[67] Ernst E Resch KL Concept of true and perceived placebo effects. Bmj (1995) 311(7004):551–3. 10.1136/bmj.311.7004.551 PMC2550609 7663213

[68] de Craen AJ Tijssen JG de Gans J Kleijnen J Placebo effect in the acute treatment of migraine: subcutaneous placebos are better than oral placebos. J Neurol (2000) 247(3):183–8. 10.1007/s004150050560 10787112

[69] Kaptchuk TJ Stason WB Davis RB Legedza AR Schnyer RN Kerr CE Sham device v inert pill: randomised controlled trial of two placebo treatments. Bmj (2006) 332(7538):391–7. 10.1136/bmj.38726.603310.55 PMC1370970 16452103

[70] de Craen AJ Moerman DE Heisterkamp SH Tytgat GN Tijssen JG Kleijnen J Placebo effect in the treatment of duodenal ulcer. Br J Clin Pharmacol (1999) 48(6):853–60. 10.1046/j.1365-2125.1999.00094.x PMC2014313 10594490

[71] Pitz M Cheang M Bernstein CN Defining the predictors of the placebo response in irritable bowel syndrome. Clin Gastroenterol Hepatol (2005) 3(3):237–47. 10.1016/S1542-3565(04)00626-3 15765443

[72] Horwitz RI Viscoli CM Berkman L Donaldson RM Horwitz SM Murray CJ Treatment adherence and risk of death after a myocardial infarction. Lancet (1990) 336(8714):542–5. 10.1016/0140-6736(90)92095-Y 1975045

[73] Sox HC Jr. Margulies I Sox CH Psychologically mediated effects of diagnostic tests. Ann Intern Med (1981) 95(6):680–5. 10.7326/0003-4819-95-6-680 7305144

[74] Colloca L Benedetti F Placebo analgesia induced by social observational learning. Pain (2009) 144(1–2):28–34. 10.1016/j.pain.2009.01.033 19278785

[75] Doering BK Rief W Utilizing placebo mechanisms for dose reduction in pharmacotherapy. Trends Pharmacol Sci (2012) 33(3):165–72. 10.1016/j.tips.2011.12.001 22284159

[76] Greenberg LM Roth S Differential effects of abrupt versus gradual withdrawal of chlorpromazine in hospitalized chronic schizophrenic patients. Am J Psychiatry (1966) 123(2):221–6. 10.1176/ajp.123.2.221 5947494

[77] Ader R Classical conditioning in the treatment of psoriasis. Cutis (2000) 66(5):370–2. 11107523

[78] Goebel MU Meykadeh N Kou W Schedlowski M Hengge UR Behavioral conditioning of antihistamine effects in patients with allergic rhinitis. Psychother Psychosom (2008) 77(4):227–34. 10.1159/000126074 18418029

[79] Ader R Mercurio MG Walton J James D Davis M Ojha V Conditioned pharmacotherapeutic effects: a preliminary study. Psychosom Med (2010) 72(2):192–7. 10.1097/PSY.0b013e3181cbd38b PMC2850283 20028830

[80] van Maanen A Meijer AM Smits MG Oort FJ Classical conditioning for preserving the effects of short melatonin treatment in children with delayed sleep: a pilot study. Nat Sci Sleep (2017) 9:67. 10.2147/NSS.S129203 28331380 PMC5352231

[81] Belcher AM Cole TO Greenblatt AD Open-label dose-extending placebos for opioid use disorder: a protocol for a randomised controlled clinical trial with methadone treatment BMJ Open 2019; 9: e026604. 10.1136/bmjopen-2018-026604 PMC6596949 31230007

[82] Exton M Schult M Donath S Strubel T Nagel E Westermann J Behavioral conditioning prolongs heart allograft survival in rats. Transplantation proceedings. (1998). 30 (5):2033. 10.1016/S0041-1345(98)00522-3 9723379

[83] Cherniack EP Would the elderly be better off if they were given more placebos? Geriatr Gerontol Int (2010) 10(2):131–7. 10.1111/j.1447-0594.2009.00580.x 20100289

[84] Whalley B Hyland ME One size does not fit all: motivational predictors of contextual benefits of therapy. Psychol Psychother (2009) 82(Pt 3):291–303. 10.1348/147608309X413275 19288979

[85] Valtorta NK Kanaan M Gilbody S Ronzi S Hanratty B Loneliness and social isolation as risk factors for coronary heart disease and stroke: systematic review and meta-analysis of longitudinal observational studies. Heart (2016) 102(13):1009. 10.1136/heartjnl-2015-308790 27091846 PMC4941172

[86] Leigh-Hunt N Bagguley D Bash K Turner V Turnbull S Valtorta N An overview of systematic reviews on the public health consequences of social isolation and loneliness. Public Health (2017) 152:57–71. 10.1016/j.puhe.2017.07.035 28915435

[87] Wang J Mann F Lloyd-Evans B Ma R Johnson S Associations between loneliness and perceived social support and outcomes of mental health problems: a systematic review. BMC Psychiatry (2018) 18(1):156. 10.1186/s12888-018-1736-5 29843662 PMC5975705

[88] Cohen S Janicki-Deverts D Turner RB Doyle WJ Does hugging provide stress-buffering social support? A study of susceptibility to upper respiratory infection and illness. Psychol Sci (2014) 26(2)135–147 10.1177/0956797614559284 PMC4323947 25526910

[89] Miller FG Kaptchuk TJ The power of context: reconceptualizing the placebo effect. J R Soc Med (2008) 101(5):222–5. 10.1258/jrsm.2008.070466 PMC2376272 18463276

[90] Weimer K Colloca L Enck P Placebo effects in psychiatry: mediators and moderators. Lancet Psychiatry (2015) 2(3):246–57. 10.1016/S2215-0366(14)00092-3 PMC4370177 25815249

[91] Peters D Understanding the placebo effect in complementary medicine: theory, Practice and Research. Churchill Livingstone London, UK (2001).

[92] Kaptchuk TJ Placebo studies and ritual theory: a comparative analysis of Navajo, acupuncture and biomedical healing. Philos Trans R Soc Lond B Biol Sci (2011) 366(1572):1849–58. 10.1098/rstb.2010.0385 PMC3130398 21576142

[93] Friesen P Mesmer, the placebo effect, and the efficacy paradox: lessons for evidence based medicine and complementary and alternative medicine. Crit Public Health (2019) 4: 435–47. 10.1080/09581596.2019.1597967

[94] Brien S Lachance L Prescott P McDermott C Lewith G Homeopathy has clinical benefits in rheumatoid arthritis patients that are attributable to the consultation process but not the homeopathic remedy: a randomized controlled clinical trial. Rheumatology (Oxford, England) (2011) 50(6):1070–82. 10.1093/rheumatology/keq234 PMC3093927 21076131

[95] Verhoef MJ Rose MS White M Balneaves LG Declining conventional cancer treatment and using complementary and alternative medicine: a problem or a challenge? Curr Oncol (Toronto, Ont.) (2008) 15(Suppl 2):s101–6. 10.3747/co.v15i0.281 PMC2528553 18769571

[96] Downey L Tyree PT Huebner CE Lafferty WE Pediatric vaccination and vaccine-preventable disease acquisition: associations with care by complementary and alternative medicine providers. Matern Child Health J (2010) 14(6):922–30. 10.1007/s10995-009-0519-5 PMC2924961 19760163

[97] Johnson SB Park HS Gross CP Yu JB Complementary medicine, refusal of conventional cancer therapy, and survival among patients with curable cancerscomplementary medicine, refusal of cancer therapy, and survival among patients with curable cancerscomplementary medicine, refusal of cancer therapy, and survival among patients with curable cancers. JAMA Oncol (2018) 4(10):1375–81. 10.1001/jamaoncol.2018.2487 PMC6233773 30027204

[98] Pickard H Responsibility without blame: empathy and the effective treatment of personality disorder. Philos Psychiatr Psychol (2011) 18(3):209. 10.1353/ppp.2011.0032 22318087 PMC3272423

[99] Friesen P Personal responsibility within health policy: unethical and ineffective. J Med Ethics (2016) 2018;44:53–58. 10.1136/medethics-2016-103478 27660291

[100] Corrigan PW On the stigma of mental illness: practical strategies for research and social change. American Psychological Association Washington, DC (2005). 10.1037/10887-000

[101] Dickson A Knussen C Flowers P Stigma and the delegitimation experience: an interpretative phenomenological analysis of people living with chronic fatigue syndrome. Psychol Health (2007) 22(7):851–67. 10.1080/14768320600976224

[102] Taft TH Keefer L Artz C Bratten J Jones MP Perceptions of illness stigma in patients with inflammatory bowel disease and irritable bowel syndrome. Qual Life Res (2011) 20(9):1391. 10.1007/s11136-011-9883-x 21424542

[103] Blease C Carel H Geraghty K Epistemic injustice in healthcare encounters: evidence from chronic fatigue syndrome. J Med Ethics (2016) 43:549–57. 10.1136/medethics-2016-103691 27920164

[104] Greco M Psychosomatic subjects and the ‘duty to be well’. Personal agency within. Econ Soc (1993) 22(3):357–72. 10.1080/03085149300000024

[105] Hossenbaccus Z White PD Views on the nature of chronic fatigue syndrome: content analysis. JRSM Short Rep (2013) 4(1):1–6. 10.1258/shorts.2012.012051 PMC3572659 23413406

[106] Nunn R It’s time to put the placebo out of our misery. BMJ (2009) 338:1015. 10.1136/bmj.b1568

[107] Crawford R Healthism and the medicalization of everyday life. Int J Health Serv (1980) 10(3):365–88. 10.2190/3H2H-3XJN-3KAY-G9NY 7419309

[108] Cheek J Healthism: a new conservatism? Qual Health Res (2008) 18(7):974–82. 10.1177/1049732308320444 18552323

[109] Friesen P Blease C Placebo effects and racial and ethnic health disparities: an unjust and underexplored connection. J Med Ethics (2018): 44:774–81. 10.1136/medethics-2018-104811 29936435

[110] Miller FG Reining in the placebo effect. Perspect Biol Med (2018) 61(3):335–48. 10.1353/pbm.2018.0046 30293972

